# Antimoniate of Quinia in Intermittents

**Published:** 1855-08

**Authors:** 


Antimoniate of Quinia in Intermittents.—Dr. La Camera, of Naples, in treating a solution of sulphate of quinia by a solution of antimoniate of potassa, has obtained a white product crystalized in needles, bitter to the taste, and soluble in hot water and in alcohol and ether. This antimoniate of quinia has given excellent results in periodical diseases, both simple and complicated with rheumatism, in sub-continued fevers of Torti, and in pernicious fevers. The dose is 0.4 grm. to 0.6 grm. during the apyrexia, and it is rarely necessary to be administered a second time.—*Ibid.*

